# Nudging Hospital Visitors Towards Stair Use, in Greece

**DOI:** 10.1007/s10935-025-00827-0

**Published:** 2025-02-08

**Authors:** Alexandros Tzikas, George Koulierakis, Konstantinos Athanasakis, Kyriakoula Merakou

**Affiliations:** 1 Department of Public Health Policy, School of Public Health, University of West Attica, Athens, Greece; 2 Laboratory of Epidemiology, Health Determinants and Well-Being, Division of Epidemiology, Prevention and Quality of Life, Department of Public Health Policy, School of Public Health, University of West Attica, Athens, Greece; 3 Laboratory for Health Technology Assessment, Division of Health Systems and Policy, Department of Public Health Policy, School of Public Health, University of West Attica, Athens, Greece

**Keywords:** Physical activity, Stair use, Health behaviour, Nudging interventions, Public health

## Abstract

Stair use is a physical activity that can be easily incorporated into daily routines, offering numerous health benefits. Nudges are increasingly adopted in public health interventions to promote healthy behaviours, such as physical activity. This study aimed to investigate the effectiveness of nudge-based posters in increasing stair use among hospital visitors in Athens, Greece. The posters were placed at the point-of-choice between stairs and elevators. Hospital visitors using either the stairs or elevators were observed across five phases, namely, baseline, two intervention phases, and two post-intervention phases, each lasting four days. A total of 3,071 choices between the stairs and the elevator were recorded during the study. The differences in proportions of stair users between stages was assessed using the Chi-square test. The results showed that the posters significantly increased stair use from 22.6% at baseline to 37.3% during the first intervention phase. Stair use dropped back to 22.2% during the first post-intervention phase. During the second intervention, stair use rising to 37.8%, followed by a decrease to 22.8% in the second post-intervention phase. These findings suggest that posters placed at the point-of-choice can effectively promote immediate behavioural changes, increasing stair use among hospital visitors. However, their long-term effect has yet to be verified. The simplicity, low cost, and easy applicability of posters make them a promising nudge-based intervention within hospital settings. These characteristics also support the generalization of this approach to other environments as part of public health policies aimed at promoting physical activity and improving overall population health.

## Introduction

Regular physical activity is vital for maintaining good health and provides significant protection against chronic diseases such as cardiovascular disease, obesity, type 2 diabetes, stroke, cancer (Nocon et al., 2008; Rhodes et al., 2017), and depression (Strawbridge et al., 2002). Stair climbing, a vigorous activity, is linked to lower blood pressure, weight loss, improved fitness, increased strength, and overall health (Andersen et al., 2013; Meyer, 2010), as well as reduced risk of death (Rey-Lopez et al., 2019). Recently, it has been shown that stair climbing also improves aerobic fitness, body composition, serum lipids, fasting blood glucose, and triglycerides (Michael et al., 2021). Just 7 min of daily stair climbing can reduce mortality in persons with coronary heart disease by 62% (Yu et al., 2003). Similarly, evidence highlights the advantages of stair climbing for healthy adults, as even brief daily bouts of regular stair climbing can improve cardiovascular fitness and cholesterol levels (Boreham et al., 2000) and reduce all-cause mortality (Lee & Paffenbarger, 2000). Furthermore, descending stair walking at home has been identified as an effective intervention for obese individuals, improving health and fitness outcomes (Chen et al., 2017; Chow et al., 2020).

Opting for stairs instead of elevators or escalators is an easily accessible way to increase physical activity, offering health benefits across diverse environments. Stair-climbing interventions have been implemented in various settings, including public spaces like train stations, universities, hospitals, and shopping malls, as well as in workplaces (Jennings et al., 2017; Tzikas et al., 2024). However, evidence suggests that interventions in workplaces may show inconsistent effectiveness, largely influenced by pedestrian flow within the built environment rather than the intervention itself (Puig-Ribera et al., 2019). On the other hand, public access settings, like hospitals and stations, where individuals may engage in repeated stair use throughout the day, may hold greater potential for significant health benefits due to stair use.

Despite the well-known health benefits of using stairs, the rate of stair users remains below 20% (Soler et al., 2010). Identifying effective interventions for behaviour change in general and stair use, in particular is essential for the success of public health initiatives (Michie et al., 2018). Nudges can influence the health behaviours of specific population groups through simple and cost-effective changes to their decision-making environment (Benartzi et al., 2017). Nudge theory, introduced by Thaler and Sunstein (2008) in their influential book on behavioural economics, proposes that people frequently make decisions based on heuristics, biases, and the context of choices rather than through careful rational deliberation. Thaler and Sunstein (2021, p. 8) define a nudge as “any aspect of the choice architecture that alters people’s behaviour in a predictable way without forbidding any options or significantly changing their economic incentives”.

In the field of public health, nudges are employed to encourage a variety of preventive health behaviours. They are used to influence dietary choices (Bauer & Reisch, 2019), enhance physical activity (Tzikas et al., 2024), and support medication adherence (Horne et al., 2022). Additionally, nudges promote preventive measures such as vaccination (Reñosa et al., 2021) and improve hand hygiene practices (Tzikas & Koulierakis, 2023).

To encourage stair use, the primary strategy involves nudging through signs and message-based interventions. This typically includes posters and banners featuring health, fitness, or calorie-related information, strategically placed near stairwells or at the entrances of elevators. Such interventions are implemented in a variety of everyday settings (Bellicha et al., 2015; Jennings et al., 2017; Tzikas et al., 2024).

To the best of our knowledge, this is the first study to examine a health-related behaviour, such as physical activity through stair use, using nudges in a hospital setting in Greece. More specifically, the study aimed to investigate the effectiveness of nudge-based posters in increasing stair use among hospital visitors in Athens, Greece. To explore this, two distinct types of posters were developed, each drawing on different psychological and behavioural theories. Poster #1 focused on a positively framed health message, emphasizing the benefits of physical activity to encourage preventive behaviour. Gain-framed messages have been shown to be particularly effective in promoting preventive health behaviours, such as regular exercise, by emphasizing the positive outcomes associated with engaging in such behaviours (Rothman & Salovey, 1997; Gallagher & Updegraff, 2012). Poster #2 leveraged social and health-related cues to amplify risk perception and discomfort, promoting behaviour change through emotional triggers. Social cues and fear of illness heighten risk awareness and trigger emotional responses, strongly influencing decision-making (Loewenstein et al., 2001; Hollands et al., 2016). Combining health risks with social discomfort nudges individuals toward healthier behaviours (Gallagher & Updegraff, 2012; Peters et al., 2012). The use of these two distinct strategies enabled the study to assess the relative effectiveness of different types of nudge-based posters in encouraging stair climbing.

To address this objective, four hypotheses were formulated. Hypothesis 1 predicted that Poster #1 would result in a significant increase in stair climbing compared to the baseline and the first post-intervention phase. Hypothesis 2 anticipated that Poster #2 would similarly lead to a significant increase in stair climbing compared to the baseline and the second post-intervention phase. Hypothesis 3 predicted that both Posters #1 and #2 would produce comparable increases in stair use, with no significant difference between them. Finally, Hypothesis 4 expected that the level of stair climbing during the post-intervention phases would return to baseline, indicating no significant difference from the baseline.

## Method

### Participants

The participants in the study were adult visitors who entered the hospital premises and chose either stairs or elevators to move from the ground floor to the higher floors of the building. The study excluded medical and nursing staff (i.e. individuals wearing medical or hospital attire), hospitalised patients, visitors with items larger than a handbag, those with a moving trolley, visibly pregnant visitors, and those physically incapable of climbing stairs.

### The Hospital

The study was conducted at the University Hospital “Attikon” in Athens, Greece. The particular hospital is a large medical facility with high patient traffic. The hospital has four floors and a variety of departments. The study focused on the busiest hallway, which provides access to key departments on each floor. This hallway leads to management offices, the pathology department, intensive care unit and the laboratory facilities on the first floor; cardiology, pediatrics, and the heart attack unit on the second floor; the ophthalmology department on the third floor; and the infectious diseases unit on the fourth floor. The hospital features wide, well-lit, and clearly marked staircases, offering a practical alternative to the elevators for visitors and staff. The elevators, centrally positioned within the building, are designed to serve both patients with mobility issues and the general public. The hospital’s layout accommodates a high volume of people, with clear signage directing patients to various departments.

### Procedure

The study included five phases: (1) baseline (2) first intervention, (3) first post-intervention, (4) second intervention, and (5) second post-intervention. This design reflects the approach first employed by Brownell et al. (1980). Observations focused exclusively on visitors using either the stairs or the elevator to move between floors. Only visitors ascending from the ground floor were included, while those descending or moving between non-ground-floor levels were excluded. Two observers were strategically positioned at a point on the ground floor to ensure visibility of both stair and elevator usage.

Interventions involved placing posters at the decision point in the hallway, where visitors had to choose between taking the stairs or the elevators. The first phase, baseline, involved observing stair use. During the second phase, a poster #1 was introduced as a nudging intervention to encourage stair use. The third phase was conducted after the poster #1 was removed. In the fourth phase, poster #2 was introduced as a nudging intervention to encourage stair use. The final post-intervention phase was conducted after the poster #2 was removed. Observations were recorded over four consecutive days for each phase, under the same conditions as the baseline.

### Development of Posters

**Fig. 1:**
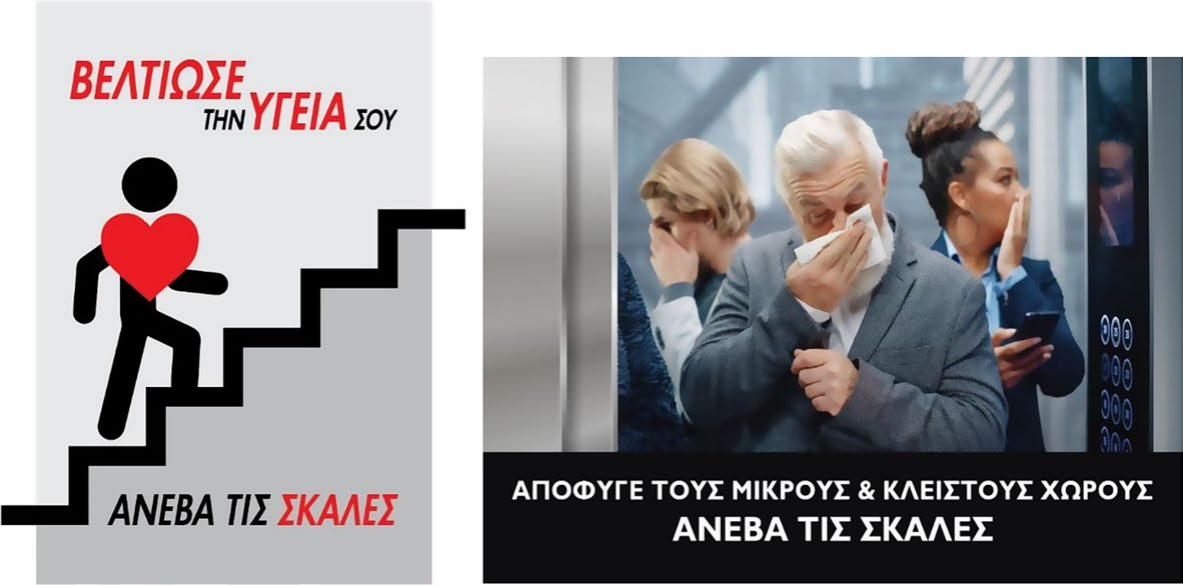
Posters to promote stair use used in this study: Poster #1 (left) and Poster #2 (right)

Poster #1 featured a positive message aimed at motivating visitors to use the stairs instead of the elevators (Fig. 1, left). It depicted a human figure-a manikin with a prominent heart in the chest area—walking up the stairs, accompanied by the phrase “Improve your health. Take the stairs”, in Greek. This message was intended to encourage visitors to choose the stairs to enhance their health. Poster #2 depicts a group of people inside an elevator (Fig. 1, right). One individual is shown wiping his nose, likely after sneezing or coughing, while the others react with visible discomfort. They instinctively cover their noses and mouths, driven by a sense of unease and concern about the potential spread of illness. This image was accompanied by the phrase “Avoid cramped, enclosed spaces. Take the stairs”, in Greek, reinforcing the idea that choosing the stairs is a healthier and safer option. Posters #1 and #2 were developed in collaboration with a graphic designer to ensure the highest quality visual presentation. The original image used in Poster #2 was purchased from Shutterstock and subsequently underwent minor editing to optimise its effectiveness. The posters were displayed in a 54 × 44 cm frame on a floor-standing tripod metal stand, positioned approximately 3 m in front of the decision point to ensure visibility.

### Monitoring Stair Use

Stair usage was observed over four consecutive days each lasting 9 h (08:00–14:00 and 17:00–20:00), during each stage of the study through direct observation. Observation of the baseline stage started on February 9, 2024. The primary researcher, along with a trained master’s student, discreetly recorded the number of visitors using either the stairs or elevators. No recording of any form of personal information of the hospital visitors was undertaken. To ensure accuracy, the observers were strategically positioned to capture participant behaviour throughout all stages of the study. Additionally, they wore civilian attire to blend in with other hospital visitors, effectively minimizing the risk of the Hawthorne effect (Berkhout et al., 2022; Chen et al., 2015). This study was conducted with the permission and approval of the hospital’s Scientific Committee (ΔΙΟΙΚ.ΕΒΔ803/21-11- 2023|15^ης^, 05/12/2023).

### Data Analysis

The proportion of visitors who used the stairs instead of the elevator was calculated by dividing the number of visitors who used the stairs by the total number of visitors who used the stairs and the elevator during the observation period. The proportions of stair users were compared across baseline, intervention periods, and the post-intervention periods and differences were assessed using the Chi-square test. Data were analysed with SPSS software and a significance level of *p* < 0.05 was applied.

## Results

A total of 3,071 decisions between the stairs and the elevator were observed during the study period (Table 1). Regarding hypothesis 1, there was a significant increase in stair use between the baseline and the first intervention phase, from 22.6 to 37.3%, χ^2^ (1) = 170.96, *p* < 0.05. Also consistent with hypothesis 1, stair use increased significantly between the baseline and the second intervention phase, from 22.6 to 37.8%, χ^2^ (1) = 183.31, *p* < 0.05. As predicted in hypothesis 3, there was no significant difference between the first and second intervention phases (37.3% vs. 37.8%, χ^2^ (1) = 0.22, *p* > 0.05). In line with hypothesis 4, stair use did not differ significantly between baseline and the post-intervention phases. Comparing baseline with post-intervention phase one (22.6% vs. 22.2%, χ^2^ (1) = 0.19, *p* > 0.05), and baseline with post-intervention phase two (22.6% vs. 22.8%, χ^2^ (1) = 0.07, *p* > 0.05) showed no significant changes.

**Table 1:** Overview of observations and percentages of stair use

Phases	Poster	No. of observations	Stair use (%)
Baseline	No	624	22.6
First intervention	Yes	598	37.3
First post- intervention	No	611	22.2
Second intervention	Yes	636	37.8
Second post- intervention	No	602	22.8

## Discussion

The present study investigated the effectiveness of nudging interventions on stair use among hospital visitors in Athens, Greece. The results showed that nudging interventions using posters with different messages had a significant impact on stair use (37.3% and 37.8%, respectively) compared to the baseline (22.6% and 22.2%, respectively), yet this effect was not sustained in the post-intervention phases (22.2% and 22.8%, respectively), where no posters were present. These findings suggest that the presence of intervention posters effectively promotes stair use, but the impact disappeared once the posters were removed. Importantly, there was no significant difference in effectiveness between the two types of posters, indicating that different nudge-based posters can produce comparable positive outcomes (Boen et al., 2010; Nomura et al., 2009; Van Hoecke et al., 2018).

The results from the two intervention phases in the current study demonstrated a substantial increase in stair use, with changes of 14.7% and 15.2% during the first and second interventions, respectively. These increases surpass the median change of 12% reported by Bellicha et al. (2015). The larger effect observed in this study could be attributed to specific contextual factors, such as the design and strategic placement of the posters, or perhaps greater responsiveness to nudging interventions within the hospital environment. In a hospital setting, people may be more attuned to health-related messaging and more likely to follow simple cues that promote healthier behaviours, such as stair use, compared to other settings (Berkhout et al., 2022). This heightened sensitivity to health prompts in a healthcare environment could explain why the intervention produced a stronger effect than what has typically been observed in other places such as worksites and public settings (Jennings et al., 2017; Tzikas et al., 2024).

The results of stair use between the baseline and post-intervention phases showed no significant changes, and the positive impact of the posters disappeared once they were removed. This suggests that there was no lasting retention effect, as the increased stair use observed during the intervention phase did not persist. This finding aligns with Titze et al. (2001), who reported that increases in stair use could not be maintained after the removal of signage. Previous studies have also demonstrated this trend, even in workplace settings where employees were repeatedly exposed to health signs (Boen et al., 2010; Buckley et al., 2015; Van den Auweele et al., 2005). However, in our study, the target group consisted exclusively of hospital visitors, rather than employees. Since visitors are a transient population, with different individuals cycling through every few days, the absence of a retention effect could be attributed to the constant turnover of people.

The posters used in this study were designed as nudge-based interventions to encourage increased physical activity. Poster #1 featured a positive message (“Improve your health. Take the stairs.“), aiming to motivate visitors to use the stairs instead of the elevator. According to Jennings et al. (2017) and Tzikas et al. (2024), the primary strategy for promoting stair use through nudging involves message-based interventions, typically using posters with health, fitness, or calorie-related content. Many of these posters depict a human figure ascending stairs, often combined with stair banners. Studies focusing on positively framed messages have consistently reported significant effects. Rothman and Salovey (1997) found that health messages emphasizing gains are more effective for promoting preventive behaviours, while loss-framed messages are better suited for detection behaviours—a finding supported by Gallagher and Updegraff’s (2012) meta-analysis.

Poster #2 employed social and health-related cues to influence health behaviour. The image of multiple people within an elevator and the accompanying phrase “Avoid cramped, enclosed spaces. Take the stairs,” this message tapped into both social discomfort and health concerns to encourage stair use. Research on nudge-based messaging, particularly in health contexts, demonstrates that social cues and fear of illness can be effective motivators for behaviour change, as they heighten awareness of potential risks and guide individuals toward healthier choices (Hollands et al., 2016). This aligns with Loewenstein et al.‘s (2001) risk as feelings hypothesis, which suggests that emotional responses, such as fear of illness, often drive decision-making more strongly than rational evaluations. By leveraging these psychological triggers, poster #2 aligns with evidence suggesting that messages combining health risks and social discomfort can nudge individuals toward preventive behaviours (Gallagher & Updegraff, 2012; Peters et al., 2012).

The results of the current study need to be cautiously considered due to specific limitations of the study. First, since no individual characteristics were recorded, we did not analyse the effects of the posters across different age groups, genders, or body weights to see if they had varying impacts on different types of visitors. Additionally, we did not track the specific floors visitors went to, nor did we account for the density of elevator use in our design. A more detailed analysis would be needed to determine whether the intervention had a greater impact on those ascending up to two floors compared to those who ascended more. Another limitation is that we did not examine the long-term effects of increased stair use. Direct observation may introduce some bias compared to automated measurements, but it allowed us to identify and exclude individuals who could not use the stairs. Future studies should address the gaps identified in this research by exploring the potential effects of demographic factors, such as age (individuals under 60), gender (men), and other factors, such as accompanying children, rush hours and body weight, on stair usage behavior. Thus, further research should investigate how these factors influence the effectiveness of nudge-based interventions, as suggested by Webb et al. (2011). Additionally, it would be valuable to examine how the number of floors ascended impacts the effectiveness of these interventions.

### Implications for Prevention

The implications for prevention highlighted by this study are promising. Nudge-based interventions, such as strategically placed posters, present a practical and cost-effective method for encouraging physical activity in everyday settings. These simple prompts can be seamlessly integrated into environments like hospitals, where promoting healthier behaviours is crucial. By subtly guiding individuals towards more active choices, these interventions can play a key role in preventive health strategies, helping to mitigate the risks associated with sedentary lifestyles. Their scalability and ease of implementation make them a valuable addition to public health policies, offering an accessible way to promote healthier habits across diverse populations. As part of a broader preventive framework, nudges can complement existing initiatives aimed at improving overall population health.

## Conclusion

The results of this study indicate that nudging-based posters placed at the point-of-choice can effectively promote immediate behavioural changes, leading to increased stair climbing among hospital visitors. Their low cost, simplicity, and wide applicability make these posters a promising preventive measure within public health policies aimed at enhancing physical activity and improving overall population health. Given their broad reach and scalability, this approach has the potential to be implemented in various settings to further encourage physical activity and improve overall public health.
